# Factors Associated with Treatment Duration in a Trauma-Focused Community Mental Health Setting

**DOI:** 10.3390/bs15070944

**Published:** 2025-07-12

**Authors:** Jason Fly, Erika Felix, Bita Ghafoori

**Affiliations:** 1 Department of Counseling, Clinical and School Psychology, University of California, Santa Barbara, CA 93106, USA; efelix@ucsb.edu; 2 Department of Advanced Studies and Counseling, California State University, Long Beach, CA 90840, USA; bita.ghafoori@csulb.edu

**Keywords:** community mental health, trauma-focused treatment, treatment duration

## Abstract

Using the behavioral model of engagement in health services, the current study assessed client characteristics that may contribute to treatment duration in trauma-focused psychotherapy in a community clinic setting. Participants (*n* = 893) were adults ages 18–78 years old (*M* = 36.36, *SD* 12.37). Demographic data (e.g., age, income) and health profile questionnaires assessing trauma and depression symptoms were collected at intake and every three sessions thereafter to track health outcome progress. Logistic regression models assessed factors associated with treatment duration at three time points: treatment initiation (0–2 sessions), treatment engagement (3–5 sessions), and treatment sustainment (6–8 sessions). For this sample, 38.6% ended treatment at the treatment initiation phase. Lower education level and higher quality of social relationships was predictive of ending treatment. In the engagement phase, 29.2% of the remaining participants (*n* = 548) ended treatment before six sessions, but there were no predictors of ending. During the sustainment phase, 31.7% ended treatment. African American race was associated with ending at this phase. In total, 70.3% of participants ended treatment before nine sessions. Participants who remained in treatment through the sustainment phase showed significant improvement in trauma and depression symptoms at each of the previous treatment phases, providing evidence of a dose response effect. Lower education, higher quality of social relationships, and African American race were associated with leaving treatment early. Many participants ended treatment before nine sessions, but those that completed treatment experienced improvement in symptoms to sub-clinical levels.

## 1. Introduction

Treatment adherence has long been an issue for the effective delivery of mental health services. Generally, barriers to engage and retain clients are poorly understood (Barrett et al., 2008). Although treatment duration has repeatedly been linked to income, race/ethnicity, or education, efforts to address this through access to community-based clinics have not significantly increased treatment retention (Barrett et al., 2008). This has particularly been an issue for trauma-focused treatment. As such, research examining retention and dropout rates of participants in psychotherapeutic treatment for trauma has increased over the years (Najavits, 2015). To date, most inquiry has been performed as a residual to the main analysis of treatment effects in randomized controlled trials (RCTs) (Berke et al., 2019), which may not generalize to naturalistic settings. The current study seeks to elucidate client characteristics, such as age, race/ethnicity, income, education, attitudes toward treatment, self-perceived quality of life, symptom severity, and trauma type, that may contribute to treatment duration in a community clinic setting. The treatment time periods assessed include *initiation*, defined as attending an in-person intake up to two sessions; *engagement*, defined as attending three sessions up to five sessions; and *sustainment*, defined as attending six sessions up to eight sessions. The aim of the current study was to investigate factors that may predict treatment duration across a full course of treatment, regardless of treatment type, because of the eclectic modalities common in community settings. Additionally, the current study investigated the effects of attendance, defined as rates of improvement in post-traumatic stress and depression symptoms, in each treatment phase for those who completed treatment.

### 1.1. Conceptual Model for Client Characteristics of Treatment Duration

The behavioral model for engaging in health services provides the theoretical basis for exploring patient characteristics that may predict treatment initiation and retention (Andersen, 1995) and includes predisposing, enabling, and need characteristics. This model has been studied for decades and serves as a framework for conceptualizing patient engagement and providing context for which areas of life are most predictive of utilizing services (Andersen, 1995). The behavioral model was originally researched with access to medical care in mind, and focused on largely white and middle-class groups, but it has been revised and adapted in the ensuing years to incorporate more patient populations and cultural context (Andersen, 1995). Although this model emphasizes prediction of initiation of treatment in medical settings, the characteristics for accessing health care are defined broadly and can be applied in all health contexts (Andersen, 1995). The current study focuses on those predisposing, enabling, and need characteristics that may help explain duration in treatment in a trauma-focused community setting, as participants move beyond access and initiation, and into a full course of treatment. Developing an understanding of why individuals discontinue trauma treatment at different time points in real-world settings, as opposed to controlled experiments, can help with strategies for treatment retention and address potential health disparities.

#### 1.1.1. Predisposing Characteristics

Predisposing factors are a set of inherent or previously achieved attributes that influence individuals access and engagement with health services. These characteristics include demographics such as age and gender; social structure, which includes factors that improve or hinder access based on your social location (i.e., race/ethnicity, education); and health beliefs, which are defined as attitudes, values, and knowledge that influence decision-making about using health services (Andersen, 1995). Previous research suggests that age, education, income, and mental health attitudes contribute to duration in psychotherapy across diagnoses (Barrett et al., 2008). For trauma-focused research, these variables also contribute to duration.

For example, in a study of an expressive writing intervention for female survivors of sexual abuse, younger age and lower education level were each associated with shorter duration (Harte et al., 2013). Further, in a study investigating rates of dropout for clients treated with co-morbid PTSD and substance use disorder, Belleau et al. (2017) showed that less education uniquely predicted dropout from the predisposing domain. Younger age was also seen as a significant predictor of shorter duration in a study of veterans comparing Cognitive Processing Therapy (CPT) and prolonged exposure (PE) rates of treatment initiation and duration, with almost 40% of participants dropping out before completing the treatment protocols (Kehle-Forbes et al., 2016). Similarly, in an analysis comparing three PTSD treatment studies, younger age predicted shorter duration in treatment (Berke et al., 2019). This was found to be consistent across the studies, regardless of treatment type

Racial/ethnic minorities have been shown to have higher dropout rates when compared to White participants (Barrett et al., 2008). In one of the largest longitudinal studies tracking children and their parents seeking trauma-focused psychotherapy using the National Child Traumatic Stress Network core dataset, Sprang et al. (2013) examined data between 2004 and 2010 to assess prediction of premature dropout. The findings suggest that African American race predicted a shorter treatment duration (Sprang et al., 2013). Race was also a factor when comparing a group of self-identified Caucasian women with African American women in treatment for trauma symptoms due to experiencing interpersonal violence (Lester et al., 2010). In this study, 55% of African American women dropped out before completing treatment as opposed to 27% of Caucasian women.

Health beliefs and attitudes were also identified as predisposing factors related to treatment initiation and duration (Andersen, 1995), and much of the research to date has focused on military populations. Active-duty soldiers who reported more stigma related to worries about career advancement, negative beliefs about treatment, and feeling that they could handle the problem on their own were more likely to drop out (Jennings et al., 2016). Negative attitudes have been consistently shown as a barrier to engaging in treatment and treatment duration among members of the military (Kim et al., 2011), but this needs to be explored further with other trauma survivors. To date, there is little research comparing trauma type, such as military combat and intimate partner violence (IPV). In studies of IPV (Gutner et al., 2016), sexual abuse, or assault histories (Harte et al., 2013; Larsen et al., 2016), the trauma types themselves were not investigated as indicators for treatment duration. Because these varying trauma features were unexamined, how they may have influenced treatment duration was overlooked. More research is needed to determine if certain trauma types or trauma characteristics increase the likelihood of reduced time in treatment, for example, for those affected by interpersonal violence compared to other types of trauma. Given the event-specific nature of traumatic disorders, this may influence the implementation of currently available treatments.

#### 1.1.2. Enabling Characteristics

Enabling characteristics allow individuals to access health care services more easily, such as personal and family resources, social relationships, health insurance, and accessibility of services (Andersen, 1995). For this study, income, quality of social relationships, and quality of an individual’s environment were assessed. Lower income can impede seeking mental health services (Ghafoori et al., 2014). The quality of a person’s social relationships has been recognized as a potential key contributing factor to treatment adherence (Andersen, 1995). Meis et al. (2019) examined the role of social environment in treatment retention among 272 veterans and their loved ones. After controlling for individual factors that may contribute to dropout, veterans who reported high levels of social support and encouragement in treatment were two times more likely to complete a course of treatment regardless of modality (Meis et al., 2019).

#### 1.1.3. Need Characteristics

Need characteristics include self-perceptions of physical and psychological health and evaluated need, such as meeting diagnostic criteria of health conditions (Andersen, 1995). Individuals exposed to traumatic events may be more vulnerable to disengaging from treatment than other populations when considering symptom severity (Najavits, 2015). The evidence is mixed, with some trauma-focused studies showing no relationship between symptom severity and treatment duration (Belleau et al., 2017), and others showing a significant contribution (Cook et al., 2013; Grubbs et al., 2015). The current study investigated symptom severity. In the context of the variety of treatment options available in community settings, it is important to understand the potential relationship of symptom severity to time in treatment to inform treatment planning.

### 1.2. Treatment Duration in Trauma-Focused Community Mental Health Settings

Ghafoori et al. (2019a) investigated factors that may predict access and initiation of treatment within a network of community-based clinics serving low-income, racially diverse victims of violence. Of the large sample in this study, only 57% accessed treatment after an initial screening, and of this screened group, 79% initiated treatment by attending the first session of an evidence-based psychotherapy (EBP). In other words, 43% failed to access treatment, and of those screened, another 21% did not initiate treatment. An unknown number may have ended treatment after the researchers’ defined initiation of treatment of attending one session. Consistent with prior research, this study showed younger age and higher symptom severity decreased access and initiation yet did not provide evidence for other factors that may have influenced engaging in treatment over time. The current study examined this community-based setting to specify factors involved in treatment duration within the longer defined time frames of initiation, engagement, and sustainment, to better identify those characteristics that may predict duration over a full course of treatment beyond accessing and initiating.

Gaining a better understanding of the effectiveness of EBPs in a trauma-focused community setting is an important step in mitigating dropout for this population. Ghafoori et al. (2019b) assessed the influence of treatment type on symptom improvement and dropout and found that person-centered therapy had the lowest rates of dropout (41.8%) compared to prolonged exposure (49.6%), cognitive behavioral (56.8%), and eclectic therapies (61.1%). There were no significant differences in symptom improvement between the treatments, however. Expanding on these studies to learn more about factors impacting treatment duration in relation to symptom improvement can help inform specific practices during earlier stages of treatment where research shows most participants leave.

#### Current Study

More research needs to be undertaken to understand trauma-focused treatment duration in community settings in light of the mixed findings on dropout in controlled studies and the limited applicability to realistic practice. Clarifying this could help with treatment planning and increase retention for clients to receive the efficacious effects of evidence-based therapies. The current study examined predisposing, enabling, and need characteristics of clients seeking treatment in an urban, trauma-focused, community-based setting over a 5-year period. Although previous research using this data has examined dropout, factors beyond the time frames of accessing and initiating treatment were not explored (Ghafoori et al., 2019a) or the researchers investigated dropout by treatment type alone (Ghafoori et al., 2019b). Given the varying nature of psychotherapies in community settings and that no significant differences were found between treatments on symptom improvement in this setting previously, the aim of the current study was to examine participant duration across engaging and sustaining treatment, regardless of treatment type. Client treatment duration was analyzed through three stages of treatment, from an initial intake through sustainment of treatment, up to a possible eight sessions. Obtaining a better understanding of client adherence to treatment during different stages of treatment can aid in treatment planning and implementation of more effective interventions. Also, examining symptom improvement over these time frames for those that persist in treatment could have implications on the efficiency of treatment protocols used in community settings. By applying the behavioral health model, this study investigated specific treatment phases and sought to answer the following questions:

**RQ1:**  *Which factors (demographic, post-traumatic and depression symptoms, trauma type, quality of life, negative attitudes toward treatment) are associated with treatment duration at each of the three phases of treatment: initiation, engagement, and sustainment?*

**RQ2:**  *What are the effects of attendance in each treatment phase on client improvement in PTSD and depression symptoms?*

## 2. Materials and Methods

### 2.1. Participants

Participants were recruited through the Long Beach Trauma and Recovery Center (LBTRC) through a supporting grant from the state of California’s victim compensation fund. The LBTRC is a community-based mental health agency offering no-cost evidence-based mental health services for victims of violence in southern California. The state grant offers a no-cost service and travel vouchers to any of the clinics in the LBTRC network. Participants were included in the study if they were over 18 years of age and were referred to the LBTRC for psychotherapeutic services for exposure to crime as a victim, or were a family member of a victim of violence. They were also required to have an initial screening (*n* = 1018) with an LBTRC staff member, and complete a baseline set of questionnaires. Those who reported being actively psychotic, had a brain injury, had impaired cognitive functioning (*n* = 51), or were missing the initial packet of questionnaires (*n* = 74) were excluded from the study (see Appendix A).

The final sample size (*n* = 893) was on average 36.46 years of age (*SD* = 12.37; range 18–78 years). Participants identified their race/ethnicity as White (16.8%), African American (15.5%), Latino (56.4%), Asian/Pacific Islander (3.1%), Native American (0.7%), or Mixed Race/Ethnicity or Other (6.7%). Most (79.2%) identified as female. Education was reported as 48.4% high school graduate or less, and 64.8% of participants reported less than USD 12,000.00 per year of household income. Trauma types were reported as domestic violence (33.5%), sexual assault (29.9%), physical assault (9.2%), traumatic loss (9.2%), family of victim (3.6%), other crime (3.7%), shooting (3.4%), human trafficking (3.3%), non-crime related trauma (2.0%), refugee trauma (0.8%), stabbing (0.7%), and vehicular assault (0.5%).

### 2.2. Procedures

Institutional Review Board (IRB) approval was obtained through the California State University, Long Beach (CSULB). All adults over the age of 18 who enrolled in the previous study were included in the analysis beginning in April 2014 until May 2019. Participants completed an initial intake interview and questionnaire packet to assess for health outcomes, mental health attitudes, and self-perceived quality of life. Thereafter, trauma-focused treatment was initiated at session one, and the questionnaire packet was administered every third psychotherapy session. Psychotherapy sessions were administered by master-level interns or master’s students in a psychology or clinical social work program. All participants received a choice of prolonged exposure, cognitive processing, narrative exposure, cognitive behavioral, eclectic, or person-centered therapy in consultation with their therapist, and were treated for the trauma type they presented with at intake. Data were collected until session 12, although for this study, data were analyzed at the defined time of completion at session 9, due to guidance for duration of evidence-based trauma-focused treatment being 8–12 sessions (APA, 2017).

### 2.3. Measures

#### 2.3.1. Demographics

Participants were asked to identify their race/ethnicity, income (i.e., less than USD 6000, USD 6000–11,999, USD 12,000–17,999, USD 18,000–35,999, USD 36,000 or greater) and education (i.e., less than or equal to 8th grade, 9th to 11th grade, high school graduate, some college, college degree or greater). Participants identified their age, which for this study was consolidated into categories of 18–30 years old, 31–45 years old, and 46 years old or greater. Reference groups for the logistic regression analysis included White for race/ethnicity, highest income and education levels, and older age categories. Race/ethnicity was analyzed into three comparison categories including African American, Latino, and Other Race, which was consolidated from the limited number of participants endorsing the previously listed categories.

#### 2.3.2. Trauma Type

Participants were asked to identify their trauma type from the selections reported in the Participants Section. For this study, domestic violence and sexual assault were consolidated into the category of interpersonal violence and all other choices were consolidated into non-interpersonal violence, which is consistent with prior research (Krug et al., 2002). These two trauma types represent over 60% of those endorsed by participants and therefore were the main trauma types of interest in the study to test for effects of treatment duration. White et al. (2013) suggest that these trauma types be considered as unique because of the nature of the crimes, especially considering ethnicity and gender, as that study had participants who were women from minority groups who were victims of domestic violence and sexual assault. The participants from this study mostly identified as female and were over 70% African American and Hispanic/Latino/a. Non-interpersonal violence was used as the reference group for analysis.

#### 2.3.3. Treatment Phase

All variables of interest were measured at three time phases defined as (1) initiation, which is the period of scheduling and completing an in-person intake and attending up to two sessions; (2) engagement, attending three to five sessions; (3) sustainment, attending six to eight, and (4) treatment completion, by attending at least nine sessions. Treatment duration variables were created using session attendance data and completion of the questionnaire packet given at sessions three, six, and nine. Those that ended treatment were coded as one and those who continued treatment were coded as zero.

#### 2.3.4. Patient Health Questionnaire-9

The Patient Health Questionnaire-9 (PHQ-9; Kroenke et al., 2001) is a nine-item self-report measure of symptoms of depression shown to reliably detect depression symptom severity with high sensitivity and specificity (Kroenke et al., 2010). Participants were asked how often they have experienced specific symptoms within the last two weeks (e.g., “little interest or pleasure in doing things”) on a Likert scale from 0 (*not at all*) to 3 (*nearly every day*). For this study, mean scores were used with higher scores indicating higher depression symptom severity. This conversion to an equivalent mean score as opposed to a sum score was used due to excessive missing data to calculate an accurate sum score. SPSS handles missingness in mean scores differently than sum scores, allowing us to retain more of our sample. Mean scores between 0.55 and 1.00 indicate mild depression, 1.11 and 1.55, moderate depression, 1.66 and 2.11, moderately severe, and 2.22 and 3.00, severe depression (Kroenke et al., 2010). For the current sample, reliability of this measure was strong with Cronbach’s α = 0.90.

#### 2.3.5. PTSD Checklist for DSM-5

The PTSD Checklist for DSM-5 (PCL-5) is a 20-item self-report PTSD symptom instrument that has good internal consistency, strong correlations with other PTSD scales, and high diagnostic efficiency (Blevins et al., 2015). Respondents reflect on their most distressing traumatic event and rate the extent to which they have been bothered by each symptom in the past month (e.g., “Avoiding memories, thoughts, or feelings related to the stressful experience?”) using a 5-point Likert scale (0 = not at all to 4 = extremely) (Weathers et al., 2013). For this study, mean scores were used for trauma severity, with a mean of 1.65 representing the clinical cutoff for likely PTSD. Clinical cutoff was calculated using the upper limit of the recommended range of 31–33 out of a possible 80 total score for likely PTSD (Blevins et al., 2015) and converting to a mean score. Similarly to the PHQ-9 score conversion, this method was used to account for missing data preventing an accurate sum score. All PCL-5 scores were anchored to the index trauma type as reported by participants. Cronbach’s α for the current sample was 0.90.

#### 2.3.6. World Health Organization Quality of Life Brief Form

The World Health Organization Quality of Life Brief Form (WHOQOL-BREF) is a 26-item self-report questionnaire on the quality of an individual’s life. The measure has four subscales about quality of physical health (e.g., How well are you able to get around?), psychological health (e.g., How well are you able to concentrate), social relationships (e.g., How satisfied are you with the support that you get from your friends?), and environment (e.g., How safe do you feel in your daily life?) (The World Health Organization Quality of Life Group, 1998). The items are rated on a Likert scale from 1 to 5 (1 = not at all to 5 = an extreme amount), with raw subscale scores transformed into a range from 4 to 20 where higher scores indicate a better quality of life in each domain (The World Health Organization Quality of Life Group, 1998). The WHOQOL-BREF has shown strong discriminant validity in its ability to distinguish between ill and well subjects in all domains in the field test sample of participants (The World Health Organization Quality of Life Group, 1998). For this study, each subscale was used as a continuous variable in the analysis for duration. Internal consistency was good with Cronbach’s α = 0.80 for the current sample.

#### 2.3.7. Attitudes Toward Seeking Professional Psychological Help Scale—Short Form

The Attitudes Toward Seeking Professional Psychological Help Scale—Short Form (ATSPPH-SF) is a 10-item scale used to determine a client’s attitudes toward engaging in psychotherapeutic services (e.g., “If I believed I was having a mental breakdown, my first inclination would be to get professional attention.”) (Elhai et al., 2008). The scale has shown strong association with a brief measure of stigma for receiving psychological help and correlations with a measure of intention to seek mental health care (Elhai et al., 2008). Items were rated on a 4-point Likert scale ranging from 0 (Disagree) to 3 (Agree). Total scores range from 0 to 30, where higher scores represent more positive attitudes toward engaging in treatment (Elhai et al., 2008). For this study, scores were converted to mean scores for analysis. Reliability analysis showed Cronbach’s α = 0.75 for the current sample.

### 2.4. Analytic Plan

Preliminary analyses were performed to screen for missing data, outliers, and normality. Binary logistic regression models were used to assess factors associated with treatment duration rates at three different time points: initiation, engagement, and sustainment. A duration variable was created using session attendance data collected at sessions three, six, and nine with 0 = continued treatment and 1 = ended treatment. Variables were entered into the regression following the domains outlined above from the behavioral health model. In step 1, the predisposing variables of race/ethnicity, age, education, seeking help attitudes, and trauma type were entered; in step 2, enabling variables of income, quality of social relationships, and quality of environment were entered; and in step 3, the need variables of PTSD symptoms, depression symptoms, quality of physical health, and quality of psychological health were entered. Significant effects were determined by odds ratios in the context of 95% confidence intervals. Additional analysis was performed using repeated-measures analysis of variance (ANOVA) to test for potential significant improvement in mean PTSD and depression symptom scores across initiation, engagement, and sustainment phases for those that completed treatment.

All analyses were performed using IBM Statistical Package for the Social Sciences, version 26 (IBM Corp., 2018). After visual inspection of the Q-Q plots, histograms, and performing the Shapiro–Wilk test, assumptions of normality were violated for all continuous variables, except for PTSD symptoms at the time of initiating treatment. Also, the assumption of sphericity was not met for repeated-measures ANOVA, and the Huynh–Feldt correction was reported. Although the analyses are robust against these assumptions, these violations are not uncommon in clinical samples, and the results should be interpreted with this in mind.

## 3. Results

### 3.1. Initiation Phase

In this stage, 38.6% (*n* = 345) ended treatment after an intake session and before attending three sessions. Of this group, from the predisposing domain (see Table 1), education level was associated with ending treatment, with participants who completed less than high school education more likely to end treatment. Race/ethnicity, age, trauma type, and attitudes toward seeking psychotherapeutic services were not related to ending treatment in this phase. From the enabling domain, higher quality of life in the area of social relationships predicted ending of treatment (see Table 2), but income and quality of life in environments were non-significant. From the need domain, PTSD or depression symptoms and quality of life in the areas of physical or psychological health (see Table 3) were not related to ending treatment.

### 3.2. Engagement Phase

In this stage, 29.2% (*n* = 160) of the remaining participants ended treatment before six sessions. There were no significant predictors of duration.

### 3.3. Sustainment Phase

During this stage, 31.7% (*n* = 123) ended treatment before nine sessions. African American race/ethnicity was associated with greater likelihood of ending treatment from the predisposing domain. No significant predictors of duration were found in the enabling or need domains. A total of 265 participants from the original group of 893 (29.7%) that initiated treatment completed nine sessions, showing that many participants ended treatment earlier.

### 3.4. Symptom Improvement

Participants who completed treatment showed significant improvements at each phase of treatment, with decreased PTSD symptom mean scores from initiation, engagement, sustainment phases, and completion (F(2.49, 746.35) = 150.79, *p* < 0.001, ηp^2^ = 0.33). Depression symptom mean scores also significantly decreased consistently throughout the treatment phases (F(2.68, 761.58) = 74.32, *p* < 0.001, ηp^2^ = 0.21). A post hoc pairwise comparison using a Bonferroni correction confirmed significant mean differences in both PTSD and depression symptoms at intake compared to session three, session three compared to session six, and session six compared to completion of treatment at session nine, providing further evidence of a dose response related to treatment duration (see Table 4).

## 4. Discussion

The current study sought to clarify questions about treatment duration for trauma-focused psychotherapy, using the behavioral health model of service use (Andersen, 1995) to identify potential reasons for ending treatment at specific time points and to better inform treatment planning and implementation. Research findings have been mixed about treatment duration in psychotherapy, especially among trauma-exposed clients. By applying the behavioral health model in a community setting, the demographic characteristics studied in relation to treatment duration was expanded to include symptom severity, quality-of-life domains, mental health treatment attitudes, and trauma type to further explore reasons for treatment duration in trauma-focused psychotherapy. The current study did not assess type of treatment attended, as that was not the research focus, and has been addressed by prior research (Ghafoori et al., 2019b).

### 4.1. Initiation Phase

From the predisposing domain, education was associated with ending treatment and is consistent with prior trauma research (Belleau et al., 2017; Harte et al., 2013). Considering related vulnerabilities such as literacy levels, there may be additional areas to consider that apply to the participants in the study (Gelberg et al., 2000). Previous research using these data showed that younger age was predictive of ending treatment before access and attending one session (Ghafoori et al., 2019a), but by expanding access and initiation to a longer phase of treatment (up to three sessions), as in the current study, this is no longer seen. Although no need characteristic variables were associated with ending treatment, Ghafoori et al. (2019a) suggest that many individuals with higher PTSD symptoms fail to access and initiate treatment, which may explain why this was not found in the early stages of treatment in the current study. From the enabling characteristic domain, the study showed that higher quality of social relationships was associated with discontinuing treatment. Those clients may have felt that they could deal with their traumas on their own with the help of family or friends, as was the case for some soldiers who reported this as a reason for terminating treatment (Hundt et al., 2018). Conversely, given the majority Latinx population of participants, sociocultural factors such as family structure and dynamics could be explored to clarify social influence on treatment duration outside of individual attitudes toward treatment or stigma, which was not found to be a factor. Collective attitudes as a factor may outweigh individual ones in an unhelpful way.

### 4.2. Engagement Phase

During this stage of treatment, no significant predictors of ending treatment were found across the behavioral model domains. This is in contrast to previous research that suggests race/ethnicity, younger age, and lower education are predictors of dropout (Belleau et al., 2017; Berke et al., 2019), yet these studies did not specify when during treatment the dropout occurred. Ghafoori et al. (2014) suggest that low-income Black males especially are less likely to utilize trauma-focused mental health services. This indicates that eligible Black participants may have been more likely not to begin treatment within the study time frames, but just as likely to continue as any other group once engaged. The current study shows that although education predicts ending treatment in the early stages, as clients progress through treatment, this predisposing factor is not significant. Other sociodemographic factors shown to increase the likelihood of ending treatment from the literature were not present in the current study.

Of the three treatment stages, the engagement phase was the lowest rate of leaving treatment. During this phase, treatment completers showed significant improvements in PTSD and depression symptoms. The largest drop in depression symptoms was seen at this stage, and although treatment completers still had clinical levels of depression, a comparison in symptom severity to those that ended treatment at this stage would provide more nuance and understanding on the effects of attendance. Given the benefits of reduced symptoms by engaging in treatment, strategies to increase treatment duration during earlier phases where attrition rates are higher are warranted. Cook et al. (2013) suggest introducing other client testimonials to socialize clients to the benefits of treatment after assessing for self-perceived levels of treatment credibility. They propose attending to risk factors to reduce attrition rates by helping to set expectations and educate clients on what a course of successful treatment would be like before initiating.

### 4.3. Sustainment Phase

African American race predicted ending treatment after attending at least six sessions, but before completing. Those that sustained to completion continued to show improvement in PTSD and depression symptoms. Ghafoori et al. (2019b) showed that there were no significant differences in depression or PTSD symptoms between those that dropped out of treatment at session six or session nine using a similar community sample. They suggest that other opportunities, obligations, or needs may motivate low-income minority groups to end treatment earlier when their symptoms are reduced. Although that interpretation was speculative and we did not measure any competing need variables, in the current study, when examining treatment completers, a significant difference is found, suggesting improvements for those who persist through treatment that may show up at sessions 7 and 8 when these variables are not measured.

In summary, the initial stages of treatment had the highest numbers of people ending treatment. The identifiable predictors of ending treatment were lower education and higher quality of social relationships. As clients progressed through treatment, none of the traditional behavioral health model characteristics showed an influence on treatment duration except for African Americans who sustained treatment for six and up to eight sessions. For those who persisted through treatment sustainment, there was a steady rate of symptom improvement where they ended with sub-clinical levels of PTSD symptoms and mild depression symptoms.

### 4.4. Strengths and Limitations

Although it is imperative to have an ample sample size in any longitudinal study to account for attrition, this is especially true when measuring treatment duration at specific time points during the course of treatment. One advantage of the study was starting with a large sample due to five years of data collection, so that meaningful comparisons could be made over the course of at least a nine-session time frame. The use of a large, diverse, community-based sample was also key in moving forward research in real-world settings where the implications can have a more applied impact. Although RCTs are an important method to determine efficacious treatments, research in naturalistic settings sheds light on the implementation of treatments and ways to adapt to the needs of the particular community. This research has provided an opportunity for more nuanced inquiry into the problem of delivery of effective psychotherapeutic services.

Despite these strengths, there are some limitations to note. The rates of attrition and the number of variables used in the models reduce the power to predict and explain duration in treatment, leaving much of the variance unaccounted for. Related to this, treatment type could not be included in the analysis because of failing missingness criteria for the five-year sample. Although treatment type did not reveal any differences in symptom improvement in a previous smaller sample of this population, this remains to be explored, as does the potential association with duration of treatment. Also, in the study, most experienced some form of interpersonal violence (i.e., domestic violence, sexual assault). White et al. (2013) have suggested that these two trauma types be treated as a unique trauma category because of the nature of the crimes committed against a largely female population. Further examination of trauma types in other clinical populations is warranted. As such, these results may not generalize to treatment duration for other clinical groups or other types of trauma such as military combat or collective traumas like natural disasters. Although this affects generalizability, the focus on this group can be a strength as it expands knowledge about serving this community. Low-income women who are racial or ethnic minorities may also be more vulnerable to ending treatment (Grote et al., 2007; White et al., 2013). In our study, participants were overwhelming female, had a low income, and identified as Hispanic or Latino/a. Given this intersectionality, a re-examination of how these characteristics may interact is justified. This may help other community-based settings promote research that can be tailored and effect change for the people they serve in a more flexible way (White et al., 2013).

All psychotherapy was provided by therapists-in-training, which may have affected participant outcome. There were no data collected on fidelity of treatment protocols or differences in experience, such as any specialized training in trauma-focused therapy, that may influence participant choice to continue treatment. Further study is warranted for any potential interactions of therapist and client match.

Finally, although this study advances effectiveness research in community settings, funding provided by a state grant removed some barriers to treatment such as cost and travel, which is not typical of most community health clinics. The results should be interpreted with this in mind.

### 4.5. Future Directions

Although the data collected in the study consisted of a comprehensive demographic profile and several health outcomes, testing of the updated behavioral health model with vulnerable populations (Gelberg et al., 2000) in more clinical samples including trauma-focused treatment is warranted. For example, immigration status would be an additional vulnerability that could impact dropout rates (Gelberg et al., 2000). Considering the largely Latinx identified sample of the study, this warrants further investigation as to how this potential vulnerability could create barriers to access treatment or additional stressors that contribute to disengagement. For this study, additional salient characteristics for the types of people served by the clinics may have provided more explanation for ending treatment, such as vulnerability for repeat victimization. To address the problems with a lack of power that may be present in future attrition-type studies, a mixed-methods or qualitative follow-up with participants may provide more insights into adhering to treatment and when effective outcomes are achieved. To achieve a clearer understanding of a true dose response effect, a comparison in symptom levels could be performed for those that end treatment and those that complete at the specific time frames. This would add more clarity around symptom severity and improvement over time. Also, comparisons by treatment type would provide a more robust claim of this effect, and not just a function of time.

Also, therapist factors could be examined as part of these approaches, such as in the working relationship and the previously mentioned training variabilities. Other therapist factors that may be considered include age, gender, and race/ethnicity as possible impediments to treatment duration, depending on the population of the participants. For example, the gender of the therapist may have treatment implications for those who have experienced interpersonal violence, such as sexual assault.

Additionally, the small number of individuals who requested services yet were not included in the study because they did not complete the intake process may be significantly larger in other community settings. Future researchers may want to include this group for any factors that may explain failure to initiate.

## 5. Conclusions

As this investigation shows, more research is needed examining treatment duration for trauma-focused psychotherapy in community settings over the course of treatment. The current study showed a clear pattern of education and higher perceived social support predicting ending treatment in the early stage. Consideration of engagement with tasks outside of therapy, as is common with some trauma-focused protocols, can inform treatment planning for potential better outcomes. Exploring clarity around helpful or unhelpful social support is also warranted. For those that sustained treatment, African Americans were more likely to end in this later stage. Investigating whether competing needs or other social determinants explain this outside of race or ethnicity can help advance addressing health disparities in a more nuanced way. Participants that persisted in and completed treatment showed significant symptom improvement in PTSD and depression throughout. For trauma-exposed individuals, this shows clear benefits of treatment retention and helps inform clinicians and researchers about potential strategies for interventions and timing when considering other factors that contribute to treatment duration. The study provides evidence of sub-clinical symptom levels at the sustainment phase, indicating a potential benefit for clients in attending between six and nine sessions, which is often shorter than some treatment protocols expect. Additionally, comparing victims of violence to other types of trauma survivors remains to be explored as a vulnerability to ending treatment. Given the client improvements found in PTSD and depression symptoms for those that completed, it is imperative to achieve a clearer understanding of factors that may influence treatment duration, so that clients may obtain the benefit of remaining in treatment for the appropriate length.

## Figures and Tables

**Table 1:** Logistic regression results for predisposing characteristics associated with treatment duration.

	Treatment Initiation (*n* = 893)				Treatment Engagement (*n* = 548)				Treatment Sustainment (*n* = 388)			
	*B*	*p*	OR	95% CI	*B*	*p*	OR	95% CI	*B*	*p*	OR	95% CI
Age												
18–30 years old	0.16	0.411	1.17	[0.80, 1.71]	0.29	0.255	1.34	[0.81, 2.21]	0.07	0.819	1.07	[0.61, 1.88]
31–45 years old	−0.04	0.819	0.96	[0.66, 1.40]	0.30	0.225	1.35	[0.83, 2.20]	−0.13	0.640	0.88	[0.51, 1.51]
Race/Ethnicity												
African American	0.29	0.249	1.34	[0.82, 2.20]	0.31	0.356	1.36	[0.71, 2.59]	1.05	**0.013**	2.85	[1.25, 6.52]
Latino	0.02	0.913	1.02	[0.67, 1.56]	−0.03	0.917	0.97	[0.57, 1.66]	0.59	0.108	1.80	[0.88, 3.70]
Other Race	0.24	0.371	1.27	[0.76, 2.13]	−0.30	0.399	0.74	[0.37, 1.49]	0.10	0.837	1.10	[0.44, 2.76]
Education												
< or = to 8th grade	0.77	**0.012**	2.14	[1.18, 3.90]	0.07	0.863	1.08	[0.47, 2.47]	0.62	0.185	1.87	[0.74, 4.70]
9th to 11th grade	0.53	**0.028**	1.70	[1.06, 2.72]	−0.12	0.704	0.89	[0.47, 1.66]	0.43	0.247	1.53	[0.75, 3.14]
HS graduate	0.22	0.396	1.25	[0.75, 2.07]	−0.42	0.230	0.66	[0.34, 1.30]	0.12	0.747	1.13	[0.53, 2.42]
Some college	0.01	0.946	1.01	[0.66, 1.54]	0.02	0.935	1.02	[0.60, 1.72]	0.02	0.958	1.02	[0.53, 1.95]
Trauma Type												
Interps. viol.	−0.01	0.948	0.99	[0.73, 1.34]	−0.10	0.619	0.91	[0.61, 1.34]	0.17	0.460	1.19	[0.75, 1.89]
MH Attitudes	−0.41	0.769	0.96	[0.73, 1.62]	−0.17	0.417	0.84	[0.56, 1.27]	−0.36	0.090	0.70	[0.46, 1.06]

**Table 2:** Logistic regression results for enabling characteristics associated with treatment duration.

	Treatment Initiation (*n* = 893)				Treatment Engagement (*n* = 548)				Treatment Sustainment (*n* = 388)			
	*B*	*p*	OR	95% CI	*B*	*p*	OR	95% CI	*B*	*p*	OR	95% CI
Income												
<USD 6000	0.44	0.120	1.55	[0.89, 2.71]	−0.29	0.386	0.75	[0.38, 1.45]	0.21	0.625	1.23	[0.54, 2.82]
USD 6000–11,999	0.30	0.315	1.35	[0.75, 2.44]	−0.72	0.053	0.49	[0.24, 1.01]	0.08	0.840	1.09	[0.46, 2.60]
USD 12,000–17,999	0.04	0.908	1.04	[0.54, 1.99]	−0.70	0.083	0.50	[0.22, 1.10]	−0.38	0.445	0.68	[0.26, 1.82]
USD 18,000–35,999	0.09	0.790	1.09	[0.58, 2.04]	−0.30	0.438	0.75	[0.35, 1.57]	−0.02	0.974	0.99	[0.39, 2.46]
Quality of Life												
Social Relationships	0.18	**0.025**	1.19	[1.02, 1.39]	0.01	0.902	1.01	[0.82, 1.26]	0.22	0.089	1.24	[0.97, 1.60]
Environmental	−0.19	0.102	0.83	[0.66, 1.04]	−0.01	0.943	0.99	[0.73, 1.35]	0.14	0.449	1.15	[0.80, 1.66]

Note: All quality-of-life variables were assessed at intake.

**Table 3:** Logistic regression results for need characteristics associated with treatment duration.

	Treatment Initiation (*n* = 893)				Treatment Engagement (*n* = 548)				Treatment Sustainment (*n* = 388)			
	*B*	*p*	OR	95% CI	*B*	*p*	OR	95% CI	*B*	*p*	OR	95% CI
Depression Symptoms	0.05	0.742	1.05	[0.78, 1.41]	−0.19	0.348	0.83	[0.56, 1.23]	−0.33	0.112	0.72	[0.48, 1.08]
Trauma Symptoms	−0.04	0.694	0.960	[0.78, 1.18]	0.03	0.842	1.03	[0.75, 1.42]	0.23	0.214	1.24	[0.88, 1.75]
Quality of Life												
Physical Health	−0.11	0.372	0.90	[0.70, 1.34]	−0.03	0.852	0.97	[0.72, 1.32]	0.12	0.482	1.13	[0.80, 1.60]
Psychological Health	0.06	0.506	1.06	[0.89, 1.28]	−0.02	0.854	0.98	[0.76, 1.26]	0.12	0.383	1.13	[0.86, 1.49]

Note: All quality-of-life variables were assessed at intake. Depression and trauma symptoms were assessed at all time points.

**Table 4:** Mean differences in post-traumatic stress and depression symptoms across all treatment phases for treatment completers (*n* = 265).

	Post-Traumatic Stress Symptom		Depression Symptoms	
Treatment Phase	*M*	*SD*	*M*	*SD*
Initiation	2.35	0.87	1.54	0.75
Engagement	1.95	0.82	1.23	0.73
Sustainment	1.65	0.86	1.13	0.81
Completion	1.43	0.88	0.95	0.70

Note: All mean differences by treatment phase are significant at the *p* < 0.05 level.

**Figure A1:**
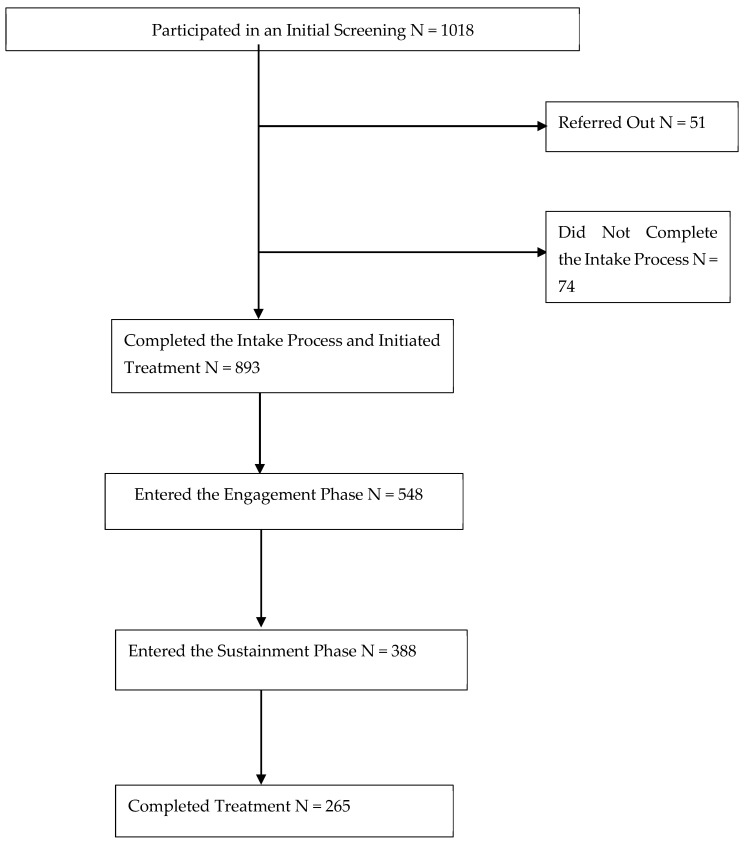
Flow chart of participants in each analysis stage.

## Data Availability

The datasets presented in this article are not readily available because participants did not give permission to share their data. Research data are not shared.
